# Supplementary figures and images for: A new molecular diagnostic tool for surveying and monitoring Triops cancriformis populations
Source: PeerJ. 2017 May 11;5:e3228. doi: 10.7717/peerj.3228 (PMC5429740; doi:10.7717/peerj.3228)

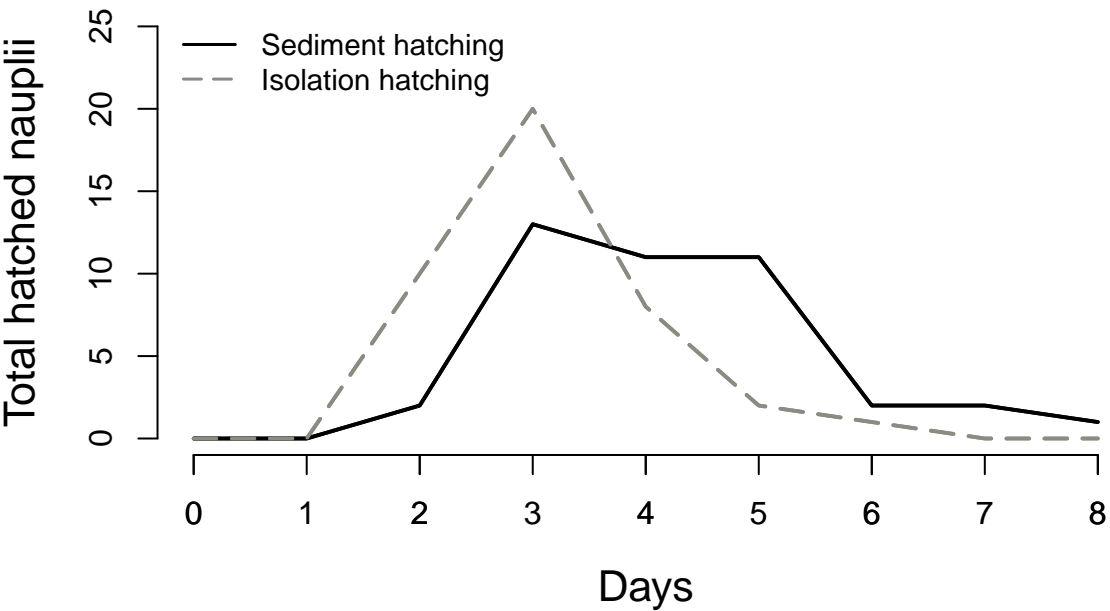

Supplement: Figure S1 — Total T. cancriformis hatchlings observed per day over the first hydroperiod for sediment and isolation hatching. Hatchling counts from all 12 sites were combined to give the daily total for each method. [file peerj-05-3228-s001.pdf]
