# Supplementary material for: A new molecular diagnostic tool for surveying and monitoring Triops cancriformis populations
Source: PeerJ. 2017 May 11;5:e3228. doi: 10.7717/peerj.3228 (PMC5429740; doi:10.7717/peerj.3228)
Supplement: Table S1 — Recorded hatchlings from the two hydroperiods and the total eggs present per site in sediment and isolation experiments are given. Hatching rates are given as the proportion of hatched eggs per site over the two hydroperiods for both methods. [file peerj-05-3228-s002.docx]

| **Hatching method** |  | **Site** | | | | | | | | | | | |
| --- | --- | --- | --- | --- | --- | --- | --- | --- | --- | --- | --- | --- | --- |
|  |  | **A** | **B** | **C** | **D** | **E** | **F** | **G** | **H** | **I** | **J** | **K** | **L** |
| **Sediment** | 1st hydroperiod | - | - | - | - | - | 2 | 27 | - | 4 | 9 | - | - |
|  | 2nd hydroperiod | - | - | - | - | - | - | 1 | - | - | - | - | - |
|  | Total eggs | 22 | 10 | 20 | 4 | 9 | 7 | 49 | 4 | 14 | 30 | 5 | 7 |
|  | Hatching rate | 0 | 0 | 0 | 0 | 0 | 0.29 | 0.57 | 0 | 0.29 | 0.3 | 0 | 0 |
| **Isolation** | 1st hydroperiod | - | - | - | 1 | - | - | 25 | - | 2 | 12 | 1 | - |
|  | 2nd hydroperiod | - | - | - | - | - | - | - | - | - | 3 | - | - |
|  | Total eggs | 22 | 11 | 15 | 2 | 7 | 1 | 58 | 15 | 13 | 49 | 15 | 6 |
|  | Hatching rate | 0 | 0 | 0 | 0.5 | 0 | 0 | 0.43 | 0 | 0.15 | 0.31 | 0.07 | 0 |
