# Supplementary material for: A new molecular diagnostic tool for surveying and monitoring Triops cancriformis populations
Source: PeerJ. 2017 May 11;5:e3228. doi: 10.7717/peerj.3228 (PMC5429740; doi:10.7717/peerj.3228)
Supplement: Table S2 — Top NCBI BLASTn hit for five short amplification and five long amplification sequences used to test the specificity of the designed primers of this study. Sequences are shown in ascending order of sample ID for each amplification region. Samples descriptions marked with ‘!’ indicate a poor quality, discarded sequence. [file peerj-05-3228-s003.docx]

| **Region** | **Sample** | **Description** | **Query length** | **Cover** | **E value** | **Ident** | **Accession** |
| --- | --- | --- | --- | --- | --- | --- | --- |
| **Short amplification** | F8 | *T. cancriformis* | 84 | 98% | 2.00E-34 | 100% | AB084514.1 |
|  | G9 | *T. cancriformis* | 84 | 98% | 2.00E-34 | 100% | AB084514.1 |
|  | I6 | *T. cancriformis* | 81 | 98% | 1.00E-32 | 100% | AB084514.1 |
|  | J7 | *T. cancriformis* | 88 | 98% | 1.00E-36 | 100% | AB084514.1 |
|  | K12 | *T. cancriformis* | 84 | 98% | 2.00E-34 | 100% | AB084514.1 |
| **Long amplification** | G28 | *T. cancriformis* | 754 | 100% | 0 | 99% | AB084514.1 |
|  | G41 | *T. cancriformis* | 498 | 100% | 0 | 99% | AB084514.1 |
|  | J15 | *T. cancriformis* | 910 | 100% | 0 | 99% | AB084514.1 |
|  | J16 | *T. cancriformis* | 910 | 100% | 0 | 99% | AB084514.1 |
|  | J17 | *!* |  |  |  |  |  |
