# Supplementary material for: A new molecular diagnostic tool for surveying and monitoring Triops cancriformis populations
Source: PeerJ. 2017 May 11;5:e3228. doi: 10.7717/peerj.3228 (PMC5429740; doi:10.7717/peerj.3228)
Supplement: Table S3 — Top NCBI BLASTn hit for 20 samples with both COI and long amplifications from the current study. Sequences are shown in ascending order of sample ID. Samples descriptions marked with ‘!’ indicate a poor quality, discarded sequence. [file peerj-05-3228-s004.docx]

|  | **COI** | | | | | | **Long amplification** | | | | | |
| --- | --- | --- | --- | --- | --- | --- | --- | --- | --- | --- | --- | --- |
| **Sample** | Description | Query length | Cover | E value | Ident | Accession | Description | Query length | Cover | E value | Ident | Accession |
| **D1** | *T. cancriformis* | 595 | 100% | 0 | 99% | JX110644.1 | *T. cancriformis* | 910 | 100% | 0 | 99% | AB084514.1 |
| **G7** | *T. cancriformis* | 561 | 100% | 0 | 99% | JX110644.1 | *!* |  |  |  |  |  |
| **G20** | *T. cancriformis* | 562 | 100% | 0 | 99% | JX110644.1 | *!* |  |  |  |  |  |
| **G25** | *T. cancriformis* | 595 | 100% | 0 | 99% | JX110644.1 | *!* |  |  |  |  |  |
| **G30** | *T. cancriformis* | 595 | 100% | 0 | 99% | JX110644.1 | *T. cancriformis* | 910 | 100% | 0 | 99% | AB084514.1 |
| **G43** | *T. cancriformis* | 595 | 100% | 0 | 99% | JX110644.1 | *T. cancriformis* | 910 | 100% | 0 | 99% | AB084514.1 |
| **G48** | *T. cancriformis* | 595 | 100% | 0 | 99% | JX110644.1 | *!* |  |  |  |  |  |
| **G53** | *T. cancriformis* | 595 | 100% | 0 | 99% | JX110644.1 | *T. cancriformis* | 910 | 100% | 0 | 99% | AB084514.1 |
| **G54** | *T. cancriformis* | 595 | 100% | 0 | 99% | JX110644.1 | *T. cancriformis* | 910 | 100% | 0 | 99% | AB084514.1 |
| **G59** | *T. cancriformis* | 595 | 100% | 0 | 99% | JX110644.1 | *T. cancriformis* | 761 | 100% | 0 | 99% | AB084514.1 |
| **I17** | *T. cancriformis* | 595 | 100% | 0 | 99% | JX110644.1 | *T. cancriformis* | 782 | 100% | 0 | 99% | AB084514.1 |
| **J1** | *T. cancriformis* | 595 | 100% | 0 | 99% | JX110644.1 | *T. cancriformis* | 910 | 100% | 0 | 99% | AB084514.1 |
| **J8** | *T. cancriformis* | 595 | 100% | 0 | 99% | JX110644.1 | *!* |  |  |  |  |  |
| **J24** | *T. cancriformis* | 593 | 100% | 0 | 99% | JX110644.1 | *!* |  |  |  |  |  |
| **J30** | *T. cancriformis* | 595 | 100% | 0 | 99% | JX110644.1 | *T. cancriformis* | 910 | 100% | 0 | 99% | AB084514.1 |
| **J31** | *T. cancriformis* | 595 | 100% | 0 | 99% | JX110644.1 | *T. cancriformis* | 910 | 100% | 0 | 99% | AB084514.1 |
| **J39** | *T. cancriformis* | 595 | 100% | 0 | 99% | JX110644.1 | *!* |  |  |  |  |  |
| **J42** | *T. cancriformis* | 507 | 100% | 0 | 99% | JX110644.1 | *!* |  |  |  |  |  |
| **J47** | *T. cancriformis* | 595 | 100% | 0 | 99% | JX110644.1 | *T. cancriformis* | 910 | 100% | 0 | 99% | AB084514.1 |
| **J51** | *T. cancriformis* | 595 | 100% | 0 | 99% | JX110644.1 | *T. cancriformis* | 910 | 100% | 0 | 99% | AB084514.1 |
