# Supplementary material for: A new molecular diagnostic tool for surveying and monitoring Triops cancriformis populations
Source: PeerJ. 2017 May 11;5:e3228. doi: 10.7717/peerj.3228 (PMC5429740; doi:10.7717/peerj.3228)
Supplement: Table S4 — Top NCBI BLASTn hit for sequences of samples with no long amplification that successfully amplified with the Folmer primers LCO1490 and HCO2198 from the current study. Sequences are shown in ascending order of E value. Sequences identified as other than T. cancriformis have Family name included in parenthesis. [file peerj-05-3228-s005.docx]

| **Sample** | **Description** | **Query length** | **Cover** | **E value** | **Ident** | **Accession** |
| --- | --- | --- | --- | --- | --- | --- |
|  |  |  |  |  |  |  |
| **G24** | *Triops cancriformis* | 595 | 100% | 0 | 99% | JX110644.1 |
| **G47** | *Triops cancriformis* | 595 | 100% | 0 | 99% | JX110644.2 |
| **J20** | *Triops cancriformis* | 405 | 100% | 0 | 99% | JX110644.1 |
| **J49** | *Triops cancriformis* | 595 | 100% | 0 | 99% | JX110644.2 |
| **K4** | *Triops cancriformis* | 595 | 100% | 0 | 99% | JX110644.3 |
| **K7** | *Triops cancriformis* | 399 | 100% | 0 | 99% | JX110644.1 |
| **K13** | *Triops cancriformis* | 595 | 100% | 0 | 99% | JX110644.2 |
| **J22** | *Cyclotella sp. (Stephanodiscaceae)* | 527 | 92% | 0 | 90% | KM202115.1 |
| **G44** | *Cyclotella sp. (Stephanodiscaceae)* | 630 | 89% | 0 | 90% | KM202115.1 |
| **J2** | *Cyclotella sp. (Stephanodiscaceae)* | 630 | 89% | 0 | 90% | KM202115.1 |
| **K5** | *Cunea thuwala (Paramoebidae)* | 526 | 100% | 0 | 89% | KP862852.1 |
| **J37** | *Cyclotella sp. (Stephanodiscaceae)* | 572 | 95% | 0 | 89% | KM202115.1 |
| **J3** | *Achlya hypogyna (Saprolegniaceae)* | 627 | 96% | 1.00E-175 | 83% | KF226724.1 |
| **G21** | *Cyclotella sp. (Stephanodiscaceae)* | 484 | 99% | 4.00E-175 | 89% | KM202115.1 |
| **L2** | *Nannochloris sp. (Coccomyxaceae)* | 578 | 93% | 1.00E-168 | 85% | KM202120.1 |
| **K11** | *Triops cancriformis* | 327 | 100% | 1.00E-164 | 100% | JX110644.1 |
| **G34** | *Homo sapiens (Hominidae)* | 326 | 100% | 3.00E-164 | 100% | NG_046602.1 |
| **J10** | *Pythium iwayamai (Pythiaceae)* | 632 | 96% | 2.00E-154 | 80% | JX397974.1 |
| **G33** | *Hartmannella vermiformis (Hartmannellidae)* | 532 | 100% | 6.00E-147 | 82% | GU828005.1 |
| **H4** | *Cyclotella sp. (Stephanodiscaceae)* | 413 | 97% | 2.00E-146 | 89% | KM202115.1 |
| **H3** | *Calosilpha brunneicollis (Silphidae)* | 302 | 100% | 7.00E-146 | 99% | HM180488.1 |
| **G14** | *Navicula minima (Naviculaceae)* | 488 | 87% | 5.00E-142 | 87% | HM449704.1 |
| **L6** | *Pythium cylindrosporum (Pythiaceae)* | 537 | 99% | 2.00E-134 | 80% | GU071824.1 |
| **I13** | *Invertebrate environmental sample* | 623 | 98% | 2.00E-134 | 77% | GU070917.1 |
| **F9** | *Invertebrate environmental sample* | 566 | 99% | 3.00E-132 | 79% | GU070917.1 |
| **J40** | *Mitrella tuberosa (Columbellidae)* | 629 | 89% | 7.00E-127 | 79% | KF643804.1 |
| **J35** | *Invertebrate environmental sample* | 633 | 88% | 3.00E-126 | 78% | GU070901.1 |
| **G19** | *Paralagenidium karlingii (Pythiaceae)* | 296 | 100% | 1.00E-112 | 91% | KC767953.1 |
| **J50** | *Roya obtusa (Mesotaeniaceae)* | 351 | 97% | 1.00E-98 | 84% | KF060943.1 |
| **A1** | *Phytophthora boehmeriae (Pythiaceae)* | 315 | 99% | 4.00E-98 | 86% | HQ261251.1 |
| **G27** | *Invertebrate environmental sample* | 323 | 99% | 5.00E-97 | 85% | GU070904.1 |
| **I12** | *Hymenoptera sp.* | 507 | 87% | 3.00E-94 | 78% | KM564452.1 |
| **J36** | *Calyptogena ponderosa endosymbiont* | 366 | 96% | 1.00E-67 | 77% | FJ899955.1 |
| **G12** | *Cyclotella sp. (Stephanodiscaceae)* | 239 | 99% | 6.00E-58 | 82% | KM202118.1 |
| **J27** | *Albugo laibachii (Albuginaceae)* | 207 | 99% | 5.00E-53 | 83% | FR832888.1 |
| **F1** | *Thiomonas sp. (Comamonadaceae)* | 226 | 99% | 7.00E-51 | 81% | LK931622.1 |
| **J29** | *Legionella oakridgensis (Legionellaceae)* | 233 | 100% | 1.00E-48 | 79% | CP004006.1 |
| **G2** | *Pinnularia neomajor (Pinnulariaceae)* | 167 | 95% | 2.00E-46 | 87% | JN418687.1 |
| **J38** | *Calyptogena ponderosa endosymbiont* | 274 | 98% | 6.00E-46 | 76% | FJ899955.1 |
| **E5** | *Chaetosoma scaritides (Chaetosomatidae)* | 116 | 98% | 2.00E-38 | 93% | EU877951.1 |
| **F2** | *Roseiflexus castenholzii (Chloroflexaceae)* | 606 | 71% | 8.00E-38 | 69% | CP000804.1 |
| **F3** | *Roseiflexus castenholzii (Chloroflexaceae)* | 419 | 91% | 1.00E-35 | 70% | CP000804.1 |
| **G36** | *Scytosiphon lomentaria (Scytosiphonaceae)* | 211 | 100% | 3.00E-31 | 75% | AB747604.1 |
| **J6** | *Echiura sp.* | 171 | 76% | 9.00E-31 | 85% | KT383422.1 |
| **I5** | *Bivalvia environmental sample* | 155 | 99% | 1.00E-29 | 81% | KP136604.1 |
| **J45** | *Durvillaea sp. (Durvillaeaceae)* | 119 | 100% | 1.00E-17 | 79% | HQ386098.1 |
| **H6** | *Legionella longbeachae (Legionellaceae)* | 141 | 56% | 5.00E-15 | 86% | FN650140.1 |
| **A5** | *Roseiflexus castenholzii  (Chloroflexaceae)* | 289 | 95% | 6.00E-14 | 68% | CP000804.1 |
| **G58** | *Pseudomonas sp. (Pseudomonadaceae)* | 304 | 32% | 2.00E-08 | 77% | KJ885299.1 |
| **G37** | *Pseudomonas sp. (Pseudomonadaceae)* | 329 | 29% | 2.00E-08 | 77% | KJ885299.1 |
| **F5** | *Streptomyces sp. (Streptomycetaceae)* | 123 | 79% | 6.00E-08 | 76% | CP015098.1 |
| **C11** | *Mesorhizobium loti (Phyllobacteriaceae)* | 202 | 38% | 2.00E-07 | 79% | CP016079.1 |
| **C2** | *Pseudonocardia dioxanivorans (Pseudonocardiaceae)* | 145 | 71% | 2.00E-06 | 74% | CP002593.1 |
| **G56** | *Micromonospora coriariae (Micromonosporaceae)* | 152 | 54% | 3.00E-05 | 76% | LT607412.1 |
| **G16** | *Roseiflexus sp. (Chloroflexaceae)* | 179 | 41% | 0.053 | 75% | CP000686.1 |
| **E4** | *Roseiflexus castenholzii (Chloroflexaceae)* | 133 | 40% | 0.64 | 80% | CP000804.1 |
