# Supplementary material for: A new molecular diagnostic tool for surveying and monitoring Triops cancriformis populations
Source: PeerJ. 2017 May 11;5:e3228. doi: 10.7717/peerj.3228 (PMC5429740; doi:10.7717/peerj.3228)
Supplement: Table S5 — Accession numbers are given for all COI sequences from Genbank. Sample ID is given for COI sequences from the current study. Species name and country of origin is also included for each sequence. [file peerj-05-3228-s006.docx]

| **Accession No. /Sample ID** | **Species** | **Country of origin** |
| --- | --- | --- |
|  |  |  |
| EF675900.1 | *T. mauritanicus* | Spain |
| DQ148291.1 | *T. cancriformis* | Italy |
| DQ369312.1 | *T. cancriformis* | Austria |
| DQ369313.1 | *T. cancriformis* | Italy |
| DQ369314.1 | *T. cancriformis* | Italy |
| DQ369315.1 | *T. cancriformis* | Sardinia |
| DQ369316.1 | *T. cancriformis* | Sicily |
| DQ369317.1 | *T. cancriformis* | Spain |
| EF189678.1 | *T. cancriformis* | Austria |
| EF675826.1 | *T. cancriformis* | Japan |
| EF675827.1 | *T. cancriformis* | Japan |
| EF675828.1 | *T. cancriformis* | Japan |
| EF675829.1 | *T. cancriformis* | Germany |
| EF675830.1 | *T. cancriformis* | Germany |
| EF675831.1 | *T. cancriformis* | Germany |
| EF675832.1 | *T. cancriformis* | Germany |
| EF675833.1 | *T. cancriformis* | Germany |
| EF675834.1 | *T. cancriformis* | Germany |
| EF675835.1 | *T. cancriformis* | Germany |
| EF675836.1 | *T. cancriformis* | Germany |
| EF675837.1 | *T. cancriformis* | Germany |
| EF675838.1 | *T. cancriformis* | Germany |
| EF675839.1 | *T. cancriformis* | Germany |
| EF675840.1 | *T. cancriformis* | Germany |
| EF675841.1 | *T. cancriformis* | Germany |
| EF675842.1 | *T. cancriformis* | Germany |
| EF675843.1 | *T. cancriformis* | Germany |
| EF675844.1 | *T. cancriformis* | Germany |
| EF675845.1 | *T. cancriformis* | Germany |
| EF675846.1 | *T. cancriformis* | Germany |
| EF675847.1 | *T. cancriformis* | Germany |
| EF675848.1 | *T. cancriformis* | Germany |
| EF675849.1 | *T. cancriformis* | Hungary |
| EF675850.1 | *T. cancriformis* | Czech Republic |
| EF675851.1 | *T. cancriformis* | Austria |
| EF675852.1 | *T. cancriformis* | Austria |
| EF675853.1 | *T. cancriformis* | Austria |
| EF675854.1 | *T. cancriformis* | England |
| EF675855.1 | *T. cancriformis* | England |
| EF675856.1 | *T. cancriformis* | England |
| EF675857.1 | *T. cancriformis* | England |
| EF675858.1 | *T. cancriformis* | England |
| EF675859.1 | *T. cancriformis* | England |
| EF675860.1 | *T. cancriformis* | England |
| EF675861.1 | *T. cancriformis* | England |
| EF675862.1 | *T. cancriformis* | Germany |
| EF675863.1 | *T. cancriformis* | Germany |
| EF675864.1 | *T. cancriformis* | Scotland |
| EF675865.1 | *T. cancriformis* | Germany |
| EF675866.1 | *T. cancriformis* | Germany |
| EF675867.1 | *T. cancriformis* | Germany |
| EF675868.1 | *T. cancriformis* | Germany |
| EF675869.1 | *T. cancriformis* | Spain |
| EF675870.1 | *T. cancriformis* | Spain |
| EF675871.1 | *T. cancriformis* | Spain |
| EF675872.1 | *T. cancriformis* | Spain |
| EF675873.1 | *T. cancriformis* | Spain |
| EF675874.1 | *T. cancriformis* | Spain |
| EF675875.1 | *T. cancriformis* | Spain |
| EF675876.1 | *T. cancriformis* | Spain |
| EF675877.1 | *T. cancriformis* | Spain |
| EF675878.1 | *T. cancriformis* | Spain |
| EF675879.1 | *T. cancriformis* | Hungary |
| EF675880.1 | *T. cancriformis* | Sicily |
| EF675881.1 | *T. cancriformis* | Sicily |
| EF675882.1 | *T. cancriformis* | Spain |
| EF675883.1 | *T. cancriformis* | Spain |
| EF675884.1 | *T. cancriformis* | Germany |
| EF675885.1 | *T. cancriformis* | Germany |
| EF675886.1 | *T. cancriformis* | Germany |
| EF675887.1 | *T. cancriformis* | Germany |
| EF675888.1 | *T. cancriformis* | Germany |
| EF675889.1 | *T. cancriformis* | Germany |
| EF675890.1 | *T. cancriformis* | Germany |
| EF675891.1 | *T. cancriformis* | Germany |
| EF675892.1 | *T. cancriformis* | Germany |
| EF675893.1 | *T. cancriformis* | Germany |
| EF675894.1 | *T. cancriformis* | Germany |
| EF675895.1 | *T. cancriformis* | Germany |
| EF675896.1 | *T. cancriformis* | Germany |
| EF675897.1 | *T. cancriformis* | Germany |
| EF675898.1 | *T. cancriformis* | Austria |
| EF675899.1 | *T. cancriformis* | Austria |
| FN691430.1 | *T. cancriformis* | United Arab Emirates |
| FN691431.2 | *T. cancriformis* | Austria |
| FN691432.2 | *T. cancriformis* | Serbia and Montenegro |
| GQ144445.1 | *T. cancriformis* | Austria |
| JN175234.1 | *T. cancriformis* | Belgium |
| JN175241.1 | *T. cancriformis* | France |
| JX110644.1 | *T. cancriformis* | Spain |
| D1_Long | *T. cancriformis* | Scotland |
| G30_Long | *T. cancriformis* | Scotland |
| G43_Long | *T. cancriformis* | Scotland |
| J47_Long | *T. cancriformis* | Scotland |
| G53_Long | *T. cancriformis* | Scotland |
| G54_Long | *T. cancriformis* | Scotland |
| G59_Long | *T. cancriformis* | Scotland |
| I17_Long | *T. cancriformis* | Scotland |
| J1_Long | *T. cancriformis* | Scotland |
| J15_Long | *T. cancriformis* | Scotland |
| J16_Long | *T. cancriformis* | Scotland |
| J30_Long | *T. cancriformis* | Scotland |
| J31_Long | *T. cancriformis* | Scotland |
| J51_Long | *T. cancriformis* | Scotland |
| G7_COI | *T. cancriformis* | Scotland |
| G20_COI | *T. cancriformis* | Scotland |
| G24_COI | *T. cancriformis* | Scotland |
| G25_COI | *T. cancriformis* | Scotland |
| G48_COI | *T. cancriformis* | Scotland |
| J8_COI | *T. cancriformis* | Scotland |
| J24_COI | *T. cancriformis* | Scotland |
| J39_COI | *T. cancriformis* | Scotland |
| J47_COI | *T. cancriformis* | Scotland |
| J49_COI | *T. cancriformis* | Scotland |
| K4_COI | *T. cancriformis* | Scotland |
| K13_COI | *T. cancriformis* | Scotland |
