# Supplementary material for: A new molecular diagnostic tool for surveying and monitoring Triops cancriformis populations
Source: PeerJ. 2017 May 11;5:e3228. doi: 10.7717/peerj.3228 (PMC5429740; doi:10.7717/peerj.3228)
Supplement: Table S6 — Successful long amplifications on extractions from the remaining unhatched T. cancriformis eggs from both sediment and isolation hatching. Shown are the total number of unhatched eggs and the number of unhatched eggs that had successful long amplifications for each site for sediment and isolation hatching. [file peerj-05-3228-s007.docx]

|  |  | **Site** | | | | | | | | | | | |
| --- | --- | --- | --- | --- | --- | --- | --- | --- | --- | --- | --- | --- | --- |
|  |  | **A** | **B** | **C** | **D** | **E** | **F** | **G** | **H** | **I** | **J** | **K** | **L** |
| **Sediment hatching** | Total unhatched eggs | 22 | 10 | 20 | 4 | 9 | 5 | 21 | 4 | 10 | 21 | 5 | 7 |
|  | Long amplifications | - | - | - | - | - | - | - | - | - | 2 | - | - |
| **Isolation hatching** | Total unhatched eggs | 22 | 11 | 15 | 1 | 7 | 1 | 33 | 15 | 11 | 34 | 14 | 6 |
|  | Long amplifications | - | - | - | - | - | - | 2 | - | - | 8 | - | - |
