# Supplementary material for: A new molecular diagnostic tool for surveying and monitoring Triops cancriformis populations
Source: PeerJ. 2017 May 11;5:e3228. doi: 10.7717/peerj.3228 (PMC5429740; doi:10.7717/peerj.3228)
Supplement: Table S7 — Time expenditure and equipment cost for each of the three egg viability estimation methods used in this study. Approximated times are for processing a single dried 20 g subsample of sediment. Times are given as time to complete each process (completion time) and the maximum time a researcher must expend to execute it (staff time). Consumables costs are based upon retail values of the consumables used. [file peerj-05-3228-s008.docx]

| **Process** | **Sediment hatching** | | **Isolation hatching** | | **DNA barcoding** | |
| --- | --- | --- | --- | --- | --- | --- |
|  | Completion time | Staff time | Completion time | Staff time | Completion time | Staff time |
| **Isolation of eggs** | 1 hr | 1 hr | 1 hr | 1 hr | 1 hr | 1 hr |
| **Observation periods** | 2 x 8 d | 2 hr 40 min | 2 x 8 d | 2 hr 40 min | - | - |
| **Drying periods** | 2 x 8 d | 20 mins | 8 d* | 20 mins | - | - |
| **DNA extraction** | - | - | - | - | 1 hr | 1 hr |
| **PCR** | - | - | - | - | 4 hours | 1 hr |
| **Gel electrophoresis** | - | - | - | - | 1 hr, 30 min | 30 min |
| **Total time taken** | 32 d, 1 hr, 10 min | 4 hr, 10 min | 24 d, 1 hr, 10 min | 4 hr, 10 min | 7 hr, 30 min | 3 hr, 30 min |
| **Consumables cost** | Very low (0.20 GBP) | | Very low (0.20 GBP) | | High (30.00 GBP) | |
| *isolation hatching only requires a single drying period between observations for completion of the method. | | | | | | |
