# Supplementary material for: A new molecular diagnostic tool for surveying and monitoring Triops cancriformis populations
Source: PeerJ. 2017 May 11;5:e3228. doi: 10.7717/peerj.3228 (PMC5429740; doi:10.7717/peerj.3228)
Supplement: Table S8 — Estimated egg bank densities (eggs/kg sediment) for the 12 sites of this study. [file peerj-05-3228-s009.docx]

| **Site** | **Eggs/20g subsample** | | | **Eggs/kg sediment** | | | **Average eggs/ kg sediment** |
| --- | --- | --- | --- | --- | --- | --- | --- |
|  | **Sediment** | **Isolation** | **DNA Barcoding** | **Sediment** | **Isolation** | **DNA Barcoding** |  |
| **A** | 22 | 22 | 18 | 1100 | 1100 | 900 | 1033.3 |
| **B** | 10 | 11 | 11 | 500 | 550 | 550 | 533.3 |
| **C** | 20 | 15 | 23 | 1000 | 750 | 1150 | 966.7 |
| **D** | 4 | 2 | 5 | 200 | 100 | 250 | 183.3 |
| **E** | 9 | 7 | 6 | 450 | 350 | 300 | 366.7 |
| **F** | 7 | 1 | 10 | 350 | 50 | 500 | 300 |
| **G** | 49 | 58 | 60 | 2450 | 2900 | 3000 | 2783.3 |
| **H** | 4 | 15 | 6 | 200 | 750 | 300 | 416.7 |
| **I** | 14 | 13 | 17 | 700 | 650 | 850 | 733.3 |
| **J** | 30 | 49 | 51 | 1500 | 2450 | 2550 | 2166.7 |
| **K** | 5 | 15 | 13 | 250 | 750 | 650 | 550 |
| **L** | 7 | 6 | 6 | 350 | 300 | 300 | 316.7 |
