# Supplementary material for: A new molecular diagnostic tool for surveying and monitoring Triops cancriformis populations
Source: PeerJ. 2017 May 11;5:e3228. doi: 10.7717/peerj.3228 (PMC5429740; doi:10.7717/peerj.3228)
Supplement: Table S9 — Viable, degraded and totally degraded eggs per kg sediment for the 12 sites of this study from the molecular method. [file peerj-05-3228-s010.docx]

| **Site** | **Egg condition/kg sediment** | | |
| --- | --- | --- | --- |
|  | **Viable** | **Degraded** | **Totally degraded** |
| **A** | 0 | 0 | 900 |
| **B** | 0 | 0 | 550 |
| **C** | 0 | 0 | 1150 |
| **D** | 50 | 0 | 200 |
| **E** | 0 | 0 | 300 |
| **F** | 50 | 0 | 450 |
| **G** | 1250 | 250 | 1500 |
| **H** | 0 | 0 | 300 |
| **I** | 150 | 50 | 650 |
| **J** | 1100 | 100 | 1350 |
| **K** | 100 | 250 | 300 |
| **L** | 0 | 0 | 300 |
